# Effects of Filler–Bitumen Ratio and Mineral Filler Characteristics on the Low-Temperature Performance of Bitumen Mastics

**DOI:** 10.3390/ma11071155

**Published:** 2018-07-06

**Authors:** Chuanfeng Zheng, Ruiming Li, Linlin Zou, Dan Lv, Yazhi Xu

**Affiliations:** College of Construction Engineering, Jilin University, Changchun 130026, China; cfzheng@jlu.edu.cn (C.Z.); lirm@mails.jlu.edu.cn (R.L.); lvdan@jlu.edu.cn (D.L.); xuyazhi00@163.com (Y.X.)

**Keywords:** bitumen mastics, mineral filler, fixed bitumen, content, meso characteristics, filler–bitumen interaction

## Abstract

This study analyzed the effects of the filler–bitumen interaction of the content and the meso powder characteristics of the mineral filler on the low-temperature performance of bitumen mastics. Control strategies for the mineral filler content (filler–bitumen ratio (*R_FB_*)) were also determined. Panjin #90 bitumen and styrene–butadiene–styrene polymer-modified bitumen were used in the experiment. Four kinds of limestone powder were used, all of which satisfy the Chinese standard for powder particle size but exhibit different meso characteristics. Each kind of limestone powder was used to prepare bitumen mastic samples under five different *R_FB_s*. The meso voids in the unit mass (*V_g_*) of the four kinds of mineral filler were tested on the basis of the principle of the Rigden void ratio. The fixed bitumen–free bitumen ratio in the bitumen mastic samples was determined using *V_g_*, bitumen density, and *R_FB_*. The low-temperature cohesive strength of the bitumen mastics was used as the control index for critical failure, whereas variation rates of bending creep stiffness at low temperature were used as the control index for fatigue failure. Results showed that the effects of the filler–bitumen interaction of the content and the meso characteristics of the mineral filler are significant and such effects are determined by the fixed bitumen–free bitumen ratio. The optimal fixed bitumen–free bitumen ratio in the bitumen mastics under two low-temperature conditions (−30 °C and −10 °C) can be determined on the basis of the influence of the fixed bitumen–free bitumen ratio on the critical and the failure control indices. Moreover, *R_FB_* can be obtained through reverse calculation. The mineral filler content can therefore be precisely controlled, which is crucial for the rational use of mineral filler and for the improvement of the pavement performance of bitumen mastics at low temperatures.

## 1. Introduction

Recent engineering applications worldwide showed that the addition of mineral filler in bitumen mixture is necessary. The application of mineral filler not only improves the viscosity of bitumen mastics but also prevents segregation in bitumen mixture during mixing, transportation, paving, and compaction. Studies demonstrated that mineral filler can significantly improve the low-temperature strength of bitumen mastics and improve the low-temperature performance of bitumen mixture [1,2,3,4,5]. Mineral filler has therefore been considered a critical component of bitumen mixture. Although mineral filler is extensively applied in bitumen mixture, factors affecting construction, such as reasonable content, meso powder characteristics, and filler-bitumen interaction, still need to be investigated. Further research on these issues is necessary for the rational application of mineral filler.

Mineral filler content is not a sensitive technical problem in terms of using the powder to increase the viscosity of bitumen mastics and preventing segregation in the mixture [6,7]. The mineral filler content becomes a sensitive factor when the low-temperature strength of bitumen mastics is considered. Studies conducted worldwide reveal that the reasonable mineral filler content in bitumen mixture remains an unsolved technical problem because the mechanism in which mineral filler affects the low-temperature strength of bitumen mastics is not uniform. The control indices used in tests are inconsistent, and factors considered during analysis vary [8,9,10].

In recent years, numerous researchers have focused on the meso powder characteristics of mineral filler and its effect on bitumen mastic strength [11,12,13,14]. The meso characteristic is between the macroscopic characteristic and microscopic characteristic. The meso powder parameters of the mineral filler include meso gradation, specific surface area, particle size, length-diameter ratio, and roundness. The meso void features of the mineral filler comprehensively reflect the multiple meso powder parameters. If the meso powder parameters of the mineral filler are substantially different, then the meso void features of the mineral filler will be significantly different [15]. There are additives to blends that act as a filler while their specific surface area, particle size properties are significantly different from the properties of a lime filler. Examples are zeolites: warm mix asphalt additives: Clinoptilolite *S_BET_* = 18 m^2^/g, zeolite Na-P1 SBET = 95 m^2^/g [16,17]. In a previous study, a particle image analysis system was used to analyze the meso powder characteristics of mineral filler (e.g., meso gradation, specific surface area, particle size, length–diameter ratio, and roundness). The results showed that all of the mineral filler samples obtained from different sources satisfied the specified requirements, but their meso powder characteristics significantly varied [18], and the mineral filler content influences the pavement performance of bitumen mastics [19]. The rational mineral filler content is therefore an important research subject. The mineral filler content is determined on the basis of bitumen pavement performance indices. The results however also vary because the control indices are different and several of the control indices lack rationality [20,21]. More importantly, the influence of the meso powder characteristics of the mineral filler is not considered at a certain mineral filler content.

Bitumen exists in bitumen mastics in two states, namely, fixed bitumen and free bitumen. The fixed bitumen–free bitumen ratio significantly affects the strength of bitumen mastics. Existing research has fully demonstrated that the content and the meso powder characteristics of mineral filler influence the fixed bitumen-free bitumen ratio in bitumen mastics. Analyzing the filler-bitumen interaction of these two factors is therefore necessary. The results of this study will provide theoretical and experimental foundation for the rational application of mineral filler in the future.

## 2. Experimental Materials

Two kinds of bitumen, which are used extensively in China, are used in this experiment. One is Panjin 90# bitumen, and the other is styrene–butadiene–styrene (SBS) polymer-modified bitumen. The penetration values of bitumen at 5 °C and 25 °C were obtained using a penetration tester according to test standards <JTG E20-2001>. A ductility tester and a softening point tester were used to test the ductility and the softening point of bitumen, respectively. The viscosities of bitumen at 60 °C and 90 °C were determined using a standard viscosity tester based on test standards <JTG E20-2001>. The viscosity tested in this study is dynamic viscosity. Table 1 shows the basic performance parameters of these two kinds of bitumen. All tests meet the standards <JTG E20-2001>.

**Figure 1:**
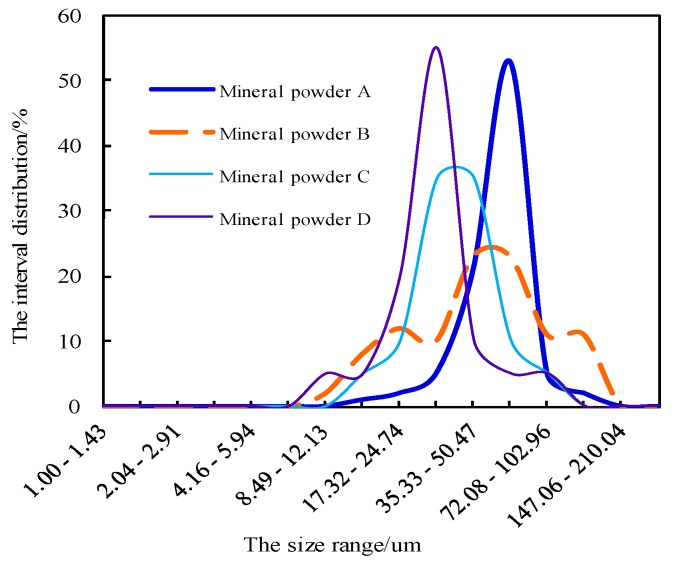
Difference in meso gradation of the four kinds of mineral powder.

**Figure 2:**
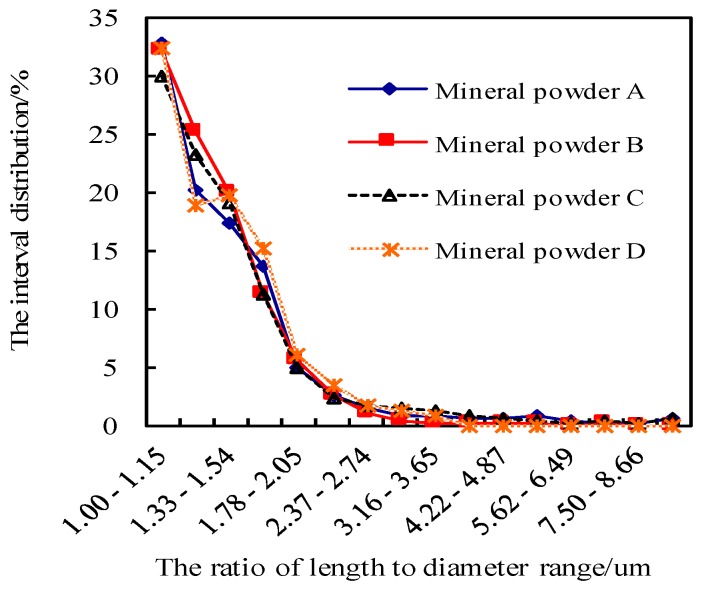
Difference in length to diameter ratio of the four kinds of mineral powder.

**Figure 3:**
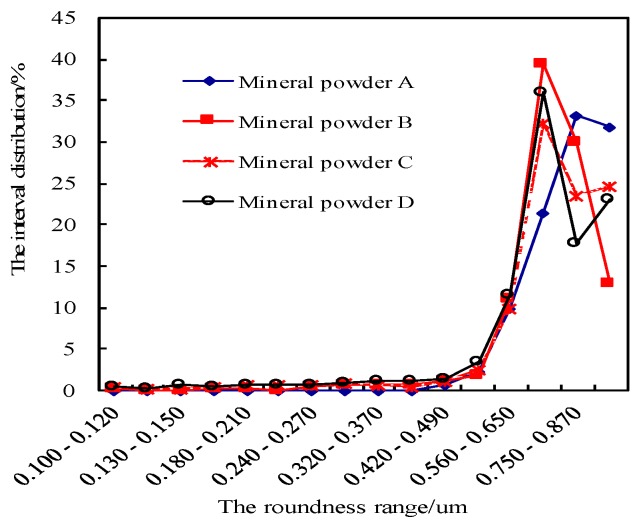
Difference in roundness of the four kinds of mineral powder.

**Figure 4:**
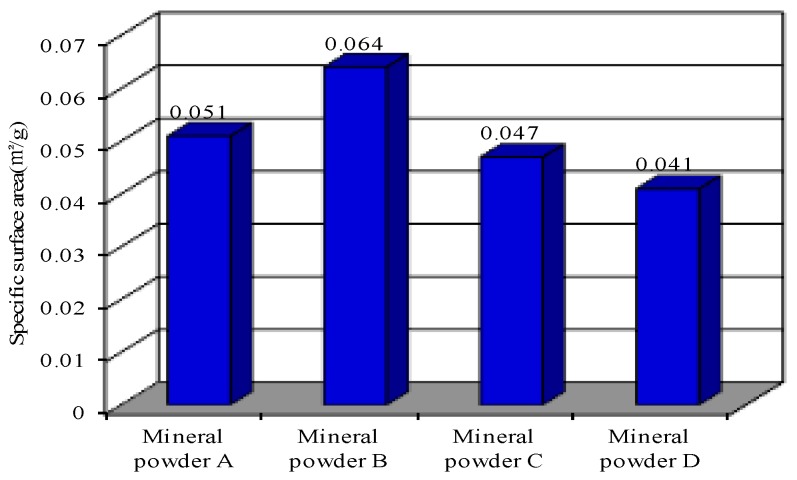
Difference in specific surface area of the four kinds of the mineral powder.

The same lithology of limestone powder was selected from 12 construction sites in Jilin Province and Liaoning Province in China. The internal analysis software of the BT-1600 particle image analysis system was used to automatically determine the gradation, specific surface area, particle size, length–diameter ratio, and roundness of the meso powder. Four kinds of mineral filler were selected by using the BT-1600 particle image analysis system, and these powders were denoted as Mineral Fillers A, B, C, and D. The size range of these mineral fillers satisfies the basic requirement specified in the “Specifications for Design of Highway Bitumen Pavement” of China (Table 2). The BT-1600 particle image analysis system however revealed that the meso gradation, specific surface area, particle size, length-diameter ratio, and roundness of meso powder are significantly different (Figure 1, Figure 2, Figure 3 and Figure 4).

The bitumen mastic samples were prepared in five filler–bitumen ratios (*R_FB_s* (the quantity of mineral filler/the quantity of bitumen); 0.6, 0.8, 1, 1.2, and 1.5). The four mineral fillers were mixed into the two bitumen samples by using a high-speed shearing machine. The high-speed shearing machine was operated at 3000 rpm. The effect of bitumen aging during the shearing process was avoided by setting the shearing temperature to 140 °C and the shearing time to 5 min. The total number of test samples is 320.

## 3. Research Method

### 3.1. Technology Roadmap

The filler–bitumen interaction of the content and the meso powder characteristics of the mineral filler are analyzed on the basis of the following research concepts in this study. First, the fixed bitumen–free bitumen ratio is quantitatively tested on the basis of the Rigden void test technique which is used to determine the filler–bitumen interaction of the content and the meso powder characteristics of mineral filler. Second, the optimal fixed bitumen–free bitumen ratio under different temperatures is determined on the basis of the control index for critical failure (low-temperature cohesive strength) and the control index for fatigue failure (variation rates of bending creep stiffness at low temperature). Lastly, the mineral filler content is calculated reversely on the basis of the filler–bitumen interaction of the content and the meso powder characteristics of the mineral filler under the premise of predetermination of the meso powder characteristics of the mineral filler.

### 3.2. Quantitative Test of the Fixed and Free Bitumens

The mineral filler is added into the bitumen to form the bitumen mastics according to the traditional principle. Bitumen mastic formation however can be understood as a process in which the bitumen is added into the mineral filler according to reverse calculation; one part of bitumen is added into the meso voids of the mineral filler to form the fixed bitumen, and the other bitumen is in free state which is called free bitumen. 

For a certain quantity of bitumen, the amount of mineral filler directly affects the mineral filler meso void, thereby affecting the proportion of fixed bitumen in bitumen mastics. The mineral filler meso void also comprehensively reflects several parameters of the meso powder characteristics, indicating that the meso powder characteristics also significantly affect the proportion of the fixed bitumen in bitumen mastics [22]. Quantitative testing of the fixed bitumen–free bitumen ratio is therefore a prerequisite for the analysis of the filler–bitumen interaction of the content and the meso powder characteristics of the mineral filler. 

Rigden voids correspond to the internal residual meso void of the mineral filler under dry and dense conditions. In the formation of bitumen mastics with a mixture of bitumen and mineral filler, fixed bitumen is formed in the meso voids. *V_g_* can be determined by utilizing technical methods to test the residual meso void of the mineral filler under dry and dense conditions; this void was then converted to the quality of mineral filler.

The volume of mineral filler after compaction is determined by this device according to the comprehensive analysis of the National Bitumen Pavement Association and the European Standards design method (EN 1097-4(24)). The actual volume of mineral filler can be determined on the basis of actual density and actual quality of mineral filler. The difference between these parameters is the residual meso void of the mineral filler. The specific test is performed according to the following procedure: 10 g of mineral filler samples is placed into containers (25 mm in diameter), and an initial compaction is applied. The samples are hammered by an impact hammer (350 g in weight, 25 mm in diameter) 100 times and the volume *V_fb_* of the mineral filler after impact compaction is measured. The actual volume *V_fs_* of 10 g of mineral filler is determined according to the quality *W_fs_* of mineral filler and the density *G_fs_* of limestone, which is used to prepare the mineral filler. The difference between *V_fb_* and *V_fs_* is the meso void of the 10 g mineral filler. The meso void of the mineral filler in unit mass *RV* can then be determined. In the preparation of bitumen mastic, the meso void of the mineral filler is filled with bitumen to form fixed bitumen. The quantity of fixed bitumen formed by mineral filler samples in unit mass is determined under the premise of definite bitumen density. For certain bitumen mastics, the quantity of mineral filler and bitumen is available. If the meso void property *RV* of the mineral filler is determined according to the method described above, then the quantity of fixed bitumen and free bitumen of mineral filler and the fixed bitumen–free bitumen ratio of bitumen mastic can be determined.

The meso void of the mineral filler in unit mass ($V_{g}$) can be expressed as follows:

(1) $$RV=V_{fb}-\frac{W_{fs}}{G_{fs}}$$

(2) $$V_{g}=\frac{RV}{W_{fs}}$$

where RV is the internal residual meso voids in the mineral filler samples after impact compaction, $V_{fb}$ is the total volume of the mineral filler particles and the internal residual meso voids after impact compaction, $W_{fs}$ is the quantity of the mineral filler samples, $G_{fs}$ is the density of limestone used to prepare the mineral filler, and $V_{g}$ is the meso voids of the mineral filler in unit mass.

### 3.3. Quantitative Testing of the Low-Temperature Cohesive Strength of Bitumen Mastics 

The performance of the bitumen mastics significantly influences the low-temperature performance of the bitumen mixture. The cracking of the bitumen mixture is manifested in the separation of the aggregates. Cracking is essentially manifested by the following two types of failure: one is bitumen mastic adhesion failure, in which the bitumen mastic is stripped from the surface of large aggregates; the other is cohesive failure, in which the bitumen mastic cracks by itself under tensile or shear load. Two kinds of failure mode are common during the onset of low-temperature damage in mixtures [23,24,25]. From the perspective of interface failure of mixtures, two kinds of strength index exist in bitumen mastics, namely, adhesive strength and cohesive strength. The results above have been accepted and recognized by numerous researchers [26,27].

Given that mineral filler directly and significantly affects the low-temperature cohesive strength of bitumen mastics, this parameter was taken in this study as the main control index for strength to analyze the filler–bitumen interaction of the content and the meso powder characteristics of mineral filler.

**Figure 5:**
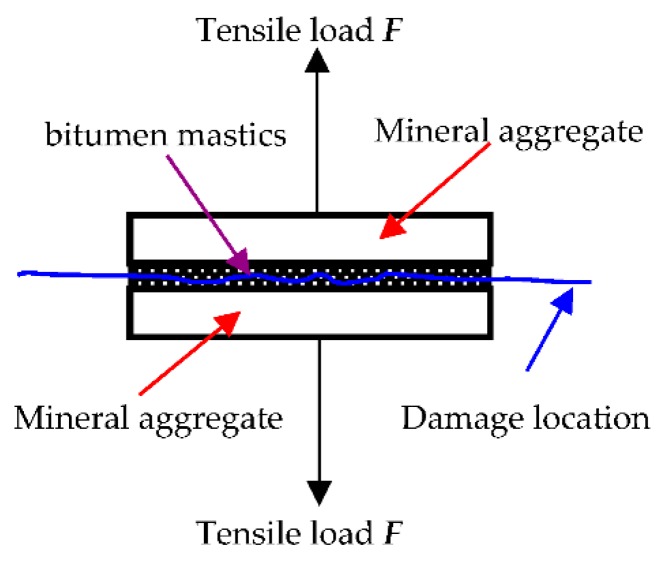
Test principle of bitumen mortar cohesive strength.

To perform this related study on the property of bitumen mastics, a quantitative testing method was developed to determine the cohesive strength of bitumen mastics. This method was authorized by Chinese invention patent (China Invention Patent Authorization Number: ZL201210359254.3) [28,29]. The low-temperature cohesive strength of bitumen mastics can be tested by using a force-measuring device, and it can be calculated according to the tensile failure load and the area of the failure interface, as shown in Formula (3). The test principle is shown in Figure 5. The loading rate in the test was 50 mm/min. The test temperatures of the specimens were controlled at −10 °C and −30 °C by a low-temperature control box.

(3) $$R_{c}\times S_{c}=F$$

where $R_{c}$ is the cohesive strength of bitumen mastics, $S_{c}$ is the failure area of the cohesive strength of bitumen mastics, and F is the failure load.

### 3.4. Rheological Effect of Bitumen Mastics

To analyze the filler–bitumen interaction of the content and the meso powder characteristics of the mineral filler more accurately and determine the control strategy for the mineral filler content, not only the low-temperature cohesive strength of bitumen was selected as the control index in this study. The bitumen mastic stiffness variation rate m, which was based on the rheological test on the bending beam of the bitumen mastics, was selected as the control index for the rheological property of bitumen mastics.

Bending beam rheological test is critical in evaluating the rheological effect of bitumen and bitumen mastics [30]. The creep stiffness variation rate m of different bitumen mastic samples was tested. A constant load (980 mN) was applied to the test specimens for 240 s in a temperature-controlled liquid (−10 °C and −30 °C). Test meets standard requirements of <JTG E20-2011>.

A large m value indicates a strong ability of bitumen and bitumen mastics for stress relaxation, and the material resistance to fatigue failure is strong as revealed by current studies [31,32].

The conventional failure and fatigue failure modes of bitumen mastics are considered in this study. The cohesive strength of bitumen mastics and the creep stiffness variation rate can be used to analyze the conventional damage and the fatigue failure, respectively.

## 4. Test Result

### 4.1. Rigden Void Test Results for the Four Mineral Fillers 

In Rigden void, the internal residual meso void in the mineral filler after impact compaction will be filled with bitumen forming the fixed bitumen. The test results for *V_g_* in Table 3 show that the amount of fixed bitumen formed by the four mineral filler samples remains significantly different when the mineral filler content (*R_FB_*) was set as the same. Although sample quantity and limestone density are the same, there is a huge difference among internal residual meso void in unit mass. Internal residual meso void in unit mass of mineral filler sample D is the maximum, 0.32, which is about three times than the minimum of mineral filler sample B, internal residual meso void in unit mass of mineral filler sample A is slightly smaller than that of mineral filler sample D that is 0.26, and internal residual meso void in unit mass of mineral filler sample C is slightly larger than that of mineral filler sample B.

The internal residual meso void in the mineral filler after impact compaction is comprehensively reflects the parameters of the meso powder characteristics, such as meso gradation, specific surface area, particle size, length–diameter ratio, and roundness. The internal residual meso void in the mineral filler after impact compaction significantly varies under the comprehensive effects of the abovementioned parameters of the meso powder characteristics. The fixed bitumen–free bitumen ratio in the bitumen mastics may therefore be different, but it ultimately affects the bitumen pavement performance.

### 4.2. Filler–Bitumen Interaction of the Content and the Meso Characteristics of the Mineral Filler

For a certain quantity of bitumen, the mixing content of the mineral filler directly affects the fixed bitumen–free bitumen ratio. The mineral filler content will therefore inevitably affect the property of bitumen mastics. The test results on *V_g_* in Table 3 demonstrated that the meso powder characteristics of the mineral filler also significantly affect the property of bitumen mastics. The content and the meso powder characteristics of the mineral filler thus exert a filler–bitumen interaction on the property of bitumen mastics.

For the application of bitumen mastics, different effects of regional environmental temperature result in significant differences in basic performance requirements of bitumen mastics. In the design stage of *R_FB_*, *R_FB_* in the bitumen mastics varies because of differences in test temperature, testing method, and control index. The current range for *R_FB_* is 0.6–1.5 according to practical application and research findings.

The quantity of the fixed bitumen and free bitumen is calculated, and the results are shown in Table 4. With the increase of *R_FB_*, the amount of mineral powder quantity increases, and the ratio of the fixed bitumen to the free bitumen also increases. The S/F (fixed/free bitumen) of mineral filler A and D is significantly higher than that of mineral filler B and C, which indicates that the performance of mineral filler A and D is better than the performance of mineral filler B and C. The combination of different *R_FB_s* and different mineral filler categories can make the fixed bitumen–free bitumen ratio equal. The SBS polymer-modified bitumen is prepared from Panjin 90# bitumen after addition of a modifier; the density of the two bitumens thus slightly differed and did not affect the quantity of the fixed and free bitumens.

### 4.3. Quantitative Test Results for Low-Temperature Cohesive Strength of Bitumen Mastics

**Figure 6:**
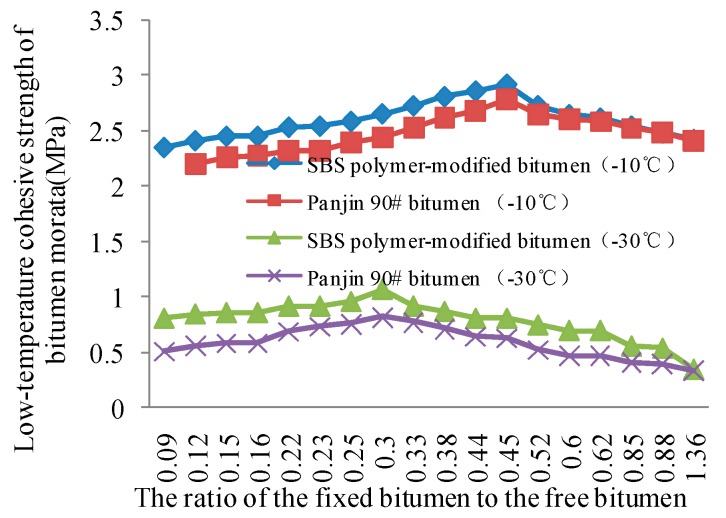
Influence of the fixed bitumen-free bitumen ratio on the low-temperature cohesive strength of bitumen mastics.

The values of the low-temperature cohesive strength of the samples were tested on the basis of the technology for the quantitative testing of the low-temperature cohesive strength of bitumen mastics; the results are shown in Figure 6. As the ratio of the fixed bitumen to the free bitumen increases, the low-temperature cohesive strength of bitumen mastics first increases and then decreases. The cohesive strength of SBS polymer-modified bitumen is higher than the cohesive strength of Panjin 90# bitumen at different temperatures, and the adhesive strength is significantly higher, when the temperature is −10 °C.

### 4.4. Test Results for the Rheological Properties of Bitumen Mastics

**Figure 7:**
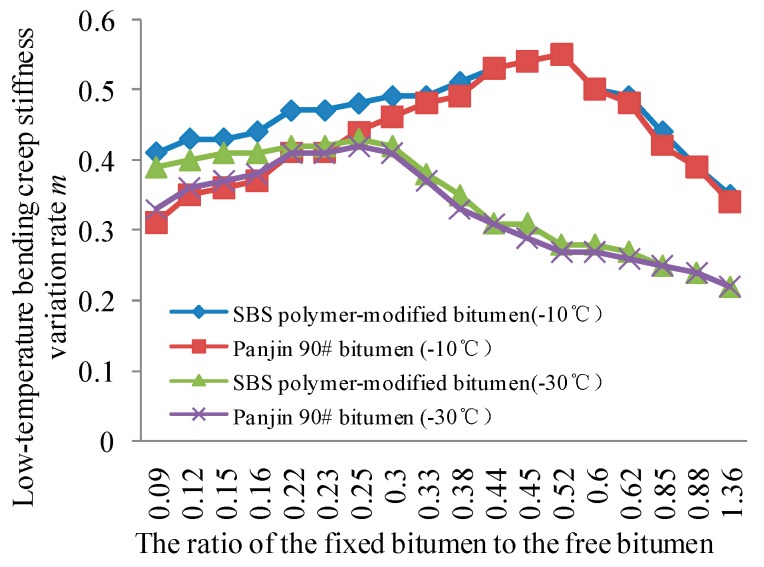
Influence of the fixed bitumen-free bitumen ratio on the low-temperature bending creep stiffness variation rate m.

The rheological properties of the bitumen mastics are tested using the bending beam rheological experiment apparatus at −30 °C and −10 °C. The resistance of the bitumen mastics to fatigue damage is characterized by the low-temperature bending creep stiffness variation rate m of the samples, and the test results are shown in Figure 7. With the ratio of the fixed bitumen to the free bitumen increasing, the asphalt increases firstly and then decreases, and the improvement is more obvious at −10 °C. And the low-temperature bending creep stiffness variation rate m of SBS polymer-modified asphalt is slightly higher than the m value of Panjin 90# bitumen.

## 5. Analysis and Discussion

### 5.1. Optimal Fixed Bitumen–Free Bitumen Ratio

This study quantitative tested the low-temperature cohesive strength and the bending creep stiffness variation rate m of bitumen mastics to determine the influence of the fixed bitumen–free bitumen ratio on the two control parameters and to analyze the influence of the filler–bitumen interaction of the content and the meso powder characteristics of the mineral filler. From the microcosmic perspective, the fixed bitumen is a component of bitumen that attaches to the surface of mineral filler particles, forming the cross-linked structure. The property of the fixed bitumen is significantly different from that of the free bitumen. The fixed bitumen–free bitumen ratio is a key internal factor influencing the bitumen mastic material and significantly influences the performance, especially at low temperature, of bitumen mastics used in roads.

As shown in Figure 6, the cohesive strength of SBS polymer-modified bitumen mastics varies within the range of 0.35–1.07 MPa and that of Panjin 90# bitumen mastics within 0.33–0.82 MPa when the range variation of the fixed bitumen–free bitumen ratio is 0.09–1.36 in extremely cold condition (−30 °C). The cohesive strength of the two kinds of bitumen mastic shows an evident peak when the fixed bitumen–free bitumen ratio is 0.3. The cohesive strength however continues to decay under this temperature condition when the ratio changed from 0.3 to 1.36. The range variation in the cohesive strength of SBS polymer-modified bitumen mastics is 2.35–2.91 MPa and that in the cohesive strength of Panjin 90# bitumen mastics is 2.20–2.78 MPa when the range variation of the fixed bitumen–free bitumen ratio is 0.09–1.36 under normal cold condition (−10 °C). The cohesive strength of the two bitumen mastics shows an evident peak when the fixed bitumen–free bitumen ratio is 0.45. The cohesive strength however continues to decay under this temperature condition when the ratio changes from 0.45 to 1.36. The fixed bitumen–free bitumen ratio significantly influences the cohesive strength of bitumen mastics when only the critical failure control index of bitumen mastics is considered. The optimal fixed bitumen–free bitumen ratio ($R_{JZ}$) in extreme and normal cold conditions is 0.3 and 0.45, respectively.

Aside from considering the control parameter for the critical failure, researchers should also analyze the influence of the fixed bitumen–free bitumen ratio on fatigue damage control parameter to determine the optimal fixed bitumen–free bitumen ratio at low temperature. Figure 7 shows that under extreme cold condition (−30 °C), the variation rate m of the low-temperature bending creep stiffness of bitumen mastics decreases, and the fatigue resistance performance of the bitumen mastics decreases at low temperature when the fixed bitumen–free bitumen ratio is greater than 0.25. Under normal cold condition (−10 °C), the variation rate m of the low-temperature bending creep stiffness of the bitumen mastics decreases, and the fatigue resistance performance of the bitumen mastics decreases at low temperature when the fixed bitumen–free bitumen ratio is greater than 0.52. Therefore, as far as the low-temperature bending creep stiffness variation rate m is concerned, the optimal fixed bitumen–free bitumen ratio in extreme and normal cold conditions is 0.25 and 0.52, respectively. 

The optimal fixed bitumen–free bitumen ratio can be determined when the critical anti-damage ability and anti-fatigue ability of the bitumen mastics can be examined simultaneously under a certain low-temperature condition. Under extreme cold condition (−30 °C), the optimal fixed bitumen–free bitumen ratio is 0.25 and 0.30, respectively, by considering the critical anti-damage ability and anti-fatigue ability of the bitumen mastics. When the fixed bitumen–free bitumen ratio is 0.25, the cohesive strength is comparable to the ratio at 0.3. However, the fixed bitumen–free bitumen ratio increases, the amount of mineral powder will increase. Therefore, taking all the influencing factors into account, the optimal fixed bitumen–free bitumen ratio is 0.25 under extreme cold condition. Under normal cold condition (−10 °C), the optimal fixed bitumen–free bitumen ratio is 0.45 and 0.52, respectively, by considering the critical anti-damage ability and anti-fatigue ability of the bitumen mastics. When the fixed bitumen–free bitumen ratio is 0.25, although the low-temperature bending creep stiffness variation rate m is the best, there is a big decrease in the cohesive strength compared with the ratio of 0.45. But when the fixed bitumen–free bitumen ratio is 0.45, the m value is also excellent. Hence, the optimal fixed bitumen–free bitumen ratio is 0.45 under normal cold condition. When the difference between the quantity of bitumen binder material and the two bitumen mastics above is large, the peak time of their cohesive strength and their bending creep stiffness variation rate will also differ. The optimal fixed bitumen–free bitumen ratio can be obtained by using the above test procedure. This is a reasonable way to determine the optimal fixed bitumen–free bitumen ratio.

### 5.2. Control Strategy for the Mineral Filler Content

**Figure 8:**
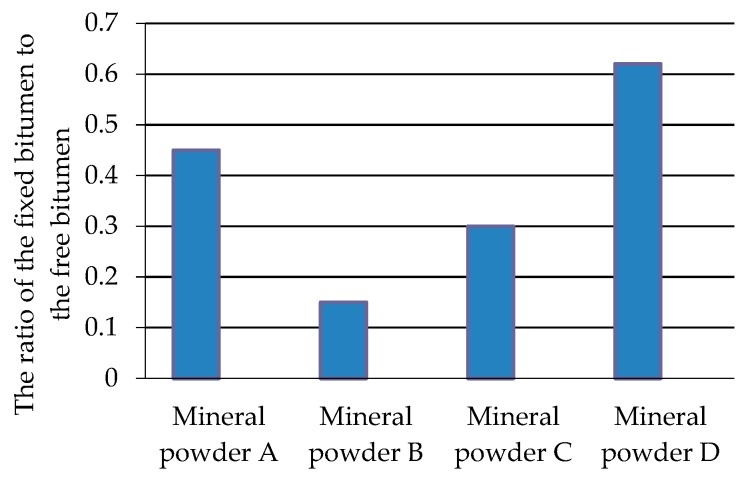
Effect of the meso powder characteristics of the mineral filler on the fixed bitumen–free bitumen ratio under the same mineral filler content.

In the present study, the fixed bitumen–free bitumen ratio would significantly vary (Figure 8; *R_FB_* is 1:1). Although the mineral filler content is the same, resulting in the difference in the pavement performance of the bitumen mastics. The ratio of mineral power B is the minimum value, which is 0.15. The maximum ratio is 0.62 for mineral power D, which is four times the minimum, and there is a huge difference between mineral power B and mineral power D. Therefore, the influential effect of the meso powder characteristics of the mineral filler should be considered when determining the mineral filler content, and the performance of mineral powder A and D is better than that of mineral powder B and C.

The SBS polymer-modified bitumen mastics is selected as an example to show the effect of the meso powder characteristics and to discuss how the mineral filler content can be determined. Under two control conditions, namely, the critical damage control index (low-temperature cohesive strength of the bitumen mastics) and the fatigue damage control index (low-temperature bending creep stiffness variation rate of the bitumen mastics), the optimal fixed bitumen–free bitumen ratio ($R_{JZ}$) under extreme cold condition (−30 °C) is 0.25, whereas 0.45 under normal cold condition (−10 °C). The mineral filler content, that is, *R_FB_* (the quantity of mineral filler/the quantity of bitumen), $R_{FB}$ is determined according to reverse calculation. In this solution, MB is the bitumen quantity, $V_{g}$ is the filler–bitumen ratio of the bitumen mastics, ρ is the meso voids of the mineral filler in unit mass, is the bitumen density, and $R_{JZ}$ is the optimal fixed bitumen–free bitumen ratio based on the critical damage control index and fatigue damage control index. The above-mentioned parameters satisfy Formula (4) which can be further simplified as Formula (5) as follows:

(4) $$\frac{M_{B}\times R_{FB}\times V_{g}\times \rho }{M_{B}-M_{B}\times R_{FB}\times V_{g}\times \rho }=R_{JZ}$$

(5) $$\frac{R_{FB}\times V_{g}\times \rho }{1-R_{FB}\times V_{g}\times \rho }=R_{JZ}$$

On the basis of the $R_{JZ}$ obtained through testing under different temperatures, the rational content of the different mineral fillers adding to the SBS polymer-modified bitumen mastics at −30 °C and −10 °C can be obtained by using Formula (5) (Table 5).

Table 5 shows that the reasonable content of the different mineral fillers varies in a certain low-temperature environment under the premise of ensuring the critical damage property and the fatigue damage property of the mastic materials. Therefore, the results determine the mineral filler content, the meso powder characteristics of mineral filler should be fully considered, and the filler–bitumen interaction of the content and the meso powder characteristics of mineral filler should be fully analyzed. 

Prior to the test, several mineral filler samples that satisfy the particle size distribution range of mineral filler in Chinese standard were collected. Mineral Fillers A and D are essential representations of the test conclusion because the values of their meso void property *V_g_* are similar to those of various mineral filler samples. Although Mineral Filler B also satisfies the Chinese standard, the meso void property differs from those of other mineral fillers, whose *V_g_* (0.11) is minimum among selected mineral filler samples. To achieve a sharp contrast, Mineral Filler B is used as a contrast sample to facilitate the analysis.

This is a method of calculating the reasonable amount of mineral filler through the optimal fixed bitumen–free bitumen ratio, which can effectively calculate the optimal amount of mineral filler, both to meet the critical anti-damage ability and to satisfy anti-fatigue ability of the bitumen mastics. It also provides a way to solve the problem that accurately control the mineral powder’s macro-dosage and provide a reasonable control strategy for mineral powder design. It is of great significance to the amount of mineral powder.

## 6. Conclusions

This study investigated the SBS polymer-modified bitumen mastics and Panjin 90# bitumen mastics which are commonly used in China. The filler–bitumen interaction of the content and the meso powder characteristics of the mineral filler are determined by the fixed bitumen–free bitumen ratio of bitumen mastics. The conclusions drawn are as follows:

(1) Although different mineral fillers satisfy the particle size control standard, the meso powder characteristics of different mineral fillers considerably vary. Results show that the meso powder characteristics of the mineral filler significantly influence the low-temperature pavement performance of bitumen mastics.
(2) The fixed bitumen–free bitumen ratio is influenced by the content and the meso powder characteristics of the mineral filler, and the filler–bitumen interaction of the content and the meso powder characteristics of mineral filler can be analyzed according to this ratio. This research proves that filler–bitumen interaction exists, and its effect is significant. The filler–bitumen interaction should be paid more attention in future research.
(3) Following previous research, this study used the low-temperature cohesive strength of bitumen mastics as the control index for critical damage and the variation rate of low-temperature bending creep stiffness as the control index for fatigue damage.
(4) Results of laboratory investigations demonstrated the effects of the fixed bitumen–free bitumen ratio on the critical damage and fatigue damage control indexes. $R_{JZ}$ under extreme cold condition (−30 °C) and normal cold condition (−10 °C) can be determined through comprehensive analysis.
(5) By considering the filler–bitumen interaction of the content and the meso voids characteristics of mineral filler, the mineral filler content can be determined reversely according to *R_JZ_* and *V_g_*. Thus, the mineral filler content can be precisely controlled, and effective control strategies for the mineral filler content can be provided. It provides a method to accurately control the mineral powder content and provide a reasonable control strategy for mineral powder design.

## Figures and Tables

**Table 1:** Basic Performance Parameters of Panjin 90# Bitumen and Styrene–Butadiene–Styrene (SBS) Polymer-modified Bitumen.

Bitumen Type	Penetration/0.1 mm		Ductility at 15 °C/cm	Softening Point/°C	Standard Viscosity/Pa·s	
	5 °C	25 °C			60 °C	90 °C
Panjin 90# bitumen	7.1	93.8	98	43.1	4120	2658
SBS polymer-modified bitumen	7.5	97.2	115	51.4	6246	3820

**Table 2:** Basic Requirements on the Size Range of Mineral Fillers in China.

Size Range (mm)	<0.6 mm	<0.15 mm	<0.075 mm
Cumulative pass rate (%)	100	90–100	75–100

**Table 3:** Test Results on the Meso Void of the Four Kinds of Mineral Filler in Unit Mass.

Mineral Filler Sample	Volume after Compaction *V_fb_* (cm^3^)	Sample Quantity (g)	Limestone Density (g/cm^3^)	Actual volume of Mineral Filler Particles *V_fs_* (cm^3^)	Internal Residual Meso Void *V_fb_* − *V_fs_* (cm^3^)	Ration of Residual Meso Void (%)	Internal Residual Meso Void in Unit Mass *V_g_* (cm^3^/g)
A	6.3	10	2.7	3.7	2.6	41.2	0.26
B	4.8	10	2.7	3.7	1.1	22.9	0.11
C	5.6	10	2.7	3.7	1.9	33.9	0.19
D	6.9	10	2.7	3.7	3.2	46.4	0.32

**Table 4:** Quantity of the Fixed and Free Bitumens Prepared by Combining Bitumen (100 g) and the Four Kinds of Mineral Filler under Five Different Filler–Bitumen Ratios (*R_FB_s*).

*R_FB_*	Mineral Filler Quantity (g)	Mineral Filler A *V_g_* = 0.26			Mineral Filler B *V_g_* = 0.11			Mineral Filler C *V_g_* = 0.19			Mineral Filler D *V_g_* = 0.32		
		S (g)	F (g)	S/F	S (g)	F (g)	S/F	S (g)	F (g)	S/F	S (g)	F (g)	S/F
0.6	60	18.72	81.28	0.23	7.92	92.08	0.09	13.68	86.32	0.16	23.04	76.96	0.30
0.8	80	24.96	75.04	0.33	10.56	89.44	0.12	18.24	81.76	0.22	30.72	69.28	0.44
1	100	31.2	68.8	0.45	13.2	86.8	0.15	22.8	77.2	0.30	38.40	61.60	0.62
1.2	120	37.44	62.56	0.60	15.84	84.16	0.19	27.36	72.64	0.38	46.08	53.92	0.85
1.5	150	46.8	53.2	0.88	19.8	80.20	0.25	34.20	65.80	0.52	57.60	42.40	1.36

S, F, and S/F represent the fixed, free, and fixed/free bitumen, respectively.

**Table 5:** Calculation Results of the Reasonable *R_FB_* in the SBS Polymer-Modified Bitumen Mastics.

Temperature (°C)	Optimum Proportion of the Fixed Bitumen–Free Bitumen Ratio	Mineral Filler Categories			
		Mineral Filler A *V_g_* = 0.26	Mineral Filler B *V_g_* = 0.11	Mineral Filler C *V_g_* = 0.19	Mineral Filler D *V_g_* = 0.32
−30	0.25	0.64	1.52	0.88	0.52
−10	0.45	0.99	2.35	1.36	0.81
